# Editorial: Deciphering cardiovascular cell heterogeneity with single cell RNA sequencing

**DOI:** 10.3389/fmed.2026.1852794

**Published:** 2026-05-08

**Authors:** Paolo Madeddu, Ngan F. Huang

**Affiliations:** 1 Bristol Medical School, Translational Health Sciences and Bristol Heart Institute, University of Bristol, Bristol, United Kingdom; 2 Department of Cardiothoracic Surgery, Stanford University, Stanford, CA, United States; 3 Stanford Cardiovascular Institute, Stanford University, Stanford, CA, United States; 4 Department of Chemical Engineering, Stanford University, Stanford, CA, United States; 5 Geriatric Research Education and Clinical Center, Veterans Affairs Palo Alto Health Care System, Palo Alto, CA, United States

**Keywords:** cardiac hemangioma, heterogeneity, immune dysregulation, lipid metabolism, small RNAs, transcriptomics, vascular atlas

Modern cell biology began in the 19th century, when Matthias Schleiden, Theodor Schwann, and later Rudolf Virchow established the idea that the cell is the fundamental unit of life. For generations, this concept has guided biological discovery and delineated the basis of clinical science. For much of this time, however, cells have been studied in aggregate, with population-level measurements masking the diversity that exists within tissues. The advent of single-cell transcriptomics has reshaped this perspective. By enabling gene expression profiling at the level of individual cells, it reveals a previously hidden spectrum of cellular states, emphasizing that even closely related cells may differ in function, regulation, and responsiveness.

In this sense, single-cell transcriptomics does not replace classical cell theory but extends it. It reinforces the centrality of the cells as the constitutive unit of organs, while highlighting their variability and context dependence. This conceptual shift is particularly relevant in cardiovascular research, where subtle differences among cells can have profound physiological and pathological consequences. The contributions collected in this Frontiers Research Topic, “*Deciphering cardiovascular cell heterogeneity with single Cell RNA sequencing*,” reflect this evolving landscape, illustrating how single-cell and integrative approaches are refining our understanding of cardiovascular biology.

The study by Li, An et al. brings a rare clinical condition into focus through the lens of single-cell analysis. By resolving the cellular composition of a neonatal cardiac hemangioma, it uncovers heterogeneity within the lesion and suggests potential mechanisms underlying its development. Its strength lies in bridging clinical observation with molecular resolution, offering insights that may inform future investigation of similar pathologies. Building upon a previously generated human vascular atlas, Avolio et al. focuses on the analysis of vasorelaxation pathways across vascular cell types. Rather than constructing the atlas itself, the study interrogates this resource to explore the distribution and potential redundancy of vasodilatory systems. This more focused approach highlights how publicly available single-cell datasets can be leveraged to address specific physiological questions, suggesting that vascular regulation may rely on overlapping and context-dependent mechanisms rather than single dominant pathways.

The study by Li, Deng et al. illustrates the increasing integration of single-cell data with computational modeling. By combining transcriptomic resolution with machine learning, it identifies lipid metabolism–related signatures associated with aortic dissection. The work moves toward a more predictive framework, with potential implications for early detection and risk stratification in vascular disease.

Besides lipid metabolism, immune dysregulation and extracellular matrix remodeling was also studied using single-cell analysis. Jin et al. examined the genetic susceptibility alongside cellular mechanisms, revealing a link between immune dysregulation, extracellular matrix remodeling, and arrhythmogenesis. The identification of SPP1^+^ macrophages as potential mediators adds a layer of cellular specificity, emphasizing the role of immune–stromal interactions in shaping cardiac electrical stability.

This Research Topic also includes a review of the regulatory framework of cardiovascular biology by focusing on tRNA-derived small RNAs (Xie et al.). Xie et al. synthesized current knowledge on their biogenesis and function, it underscores their emerging role as modulators of gene expression and their potential relevance as biomarkers or therapeutic targets.

In conclusion, these contributions reflect a field that is progressively embracing cellular resolution and molecular integration. Single-cell transcriptomics, combined with computational and conceptual advances, is not only refining our understanding of cardiovascular systems but also encouraging a more nuanced view of biological complexity. This shift, rooted in the principles of classical cell theory yet extending beyond them, continues to shape how we investigate, interpret, and ultimately approach human disease.

